# Paediatricians’ referral preference of patients with embolised intravascular foreign bodies: a survey-based study

**DOI:** 10.3402/ljm.v8i0.20495

**Published:** 2013-03-05

**Authors:** Shehla Jadoon, Milad El-Segaier, Mohammed Omar Galal

**Affiliations:** 1 Department of Paediatric Cardiology and Cardiac Surgery, PSHC, King Fahad Medical City, Riyadh, Kingdom of Saudi Arabia; 2 Department of Paediatric Cardiology, Skåne University Hospital, Lund, Sweden; 3 Department of Paediatric Cardiology, University of Essen, Essen, Germany

**Keywords:** cardiac surgery, clinical practice, transcatheter retrieval

## Abstract

**Background:**

Central line insertion is a routine procedure in medical practice. Dislodgement of lines into the vascular system is a rare complication. We noticed that paediatric health care providers (PHCP) contact the cardiac or general paediatric surgeon for extraction of dislodged lines more frequently than using the less invasive percutaneous approach.

**Aim:**

To study the referral preference of PHCP for patient with embolised intravascular foreign bodies.

**Methods:**

A questionnaire with three questions was distributed to PHCP of all paediatric subspecialties, including surgery, in two tertiary care centres. The questions were about the total number of patients seen with central line, experience with complications, and preferred specialty for removal of dislodged central lines.

**Results:**

The questionnaire was distributed to 128 professionals. The response rate was 79% (*n*=101). Incomplete answers (*n*=14) were excluded. The grades of responders were senior consultants 18%, junior consultants 38%, and residents 43%. Thirty nine percent of care providers experienced dislodgement or fragmentation of central lines. The majority (82%) prefer to refer the patients for surgical removal.

**Conclusions:**

Most PHCP in the selected hospitals prefer to refer patients with embolised foreign bodies in the vascular system for surgical removal. The local health policy should be updated for the use of the alternative percutaneous approach.

Central line insertion is a routine procedure for infusion of medications and invasive haemodynamic monitoring. Some central lines like Port-A-cath and Hickman lines are commonly used for patients receiving therapies over a longer duration. Central lines are associated with multiple problems, including infection, bleeding, functional failure, and fragmentation. Migration of a dislodged segment is a well known but rare complication (1). Broken lines are likely to migrate to the right cardiac chambers and pulmonary artery (2). Serious complications may occur if these foreign bodies are not removed.

Surgery used to be the only option for retrieving these foreign bodies from the heart and pulmonary arteries (3). However, transcatheter retrieval of intracardiac foreign bodies has been developed into a safe and easy method (4–6). The devices used include dormia baskets, snares, and tip-deflecting wires (4, 7, 8). With the introduction of snares and technical innovations, snares have evolved as the device of choice for retrieving these catheters and have replaced baskets and forceps. The use of the snare technique has simplified the procedure and increased the efficacy of retrieval, especially in small babies (9–11).

Over the last 3 years, we were able to retrieve through the transcatheter approach dislodged central lines from the right ventricle and pulmonary arteries in seven children (Fig. 1). In four of these cases, they were first referred by paediatric health care providers (PHCP) to the paediatric or cardiac surgeon for help to extract the dislodged central line. This observation prompted us to study the pattern of referral among PHCP.

**Fig. 1:**
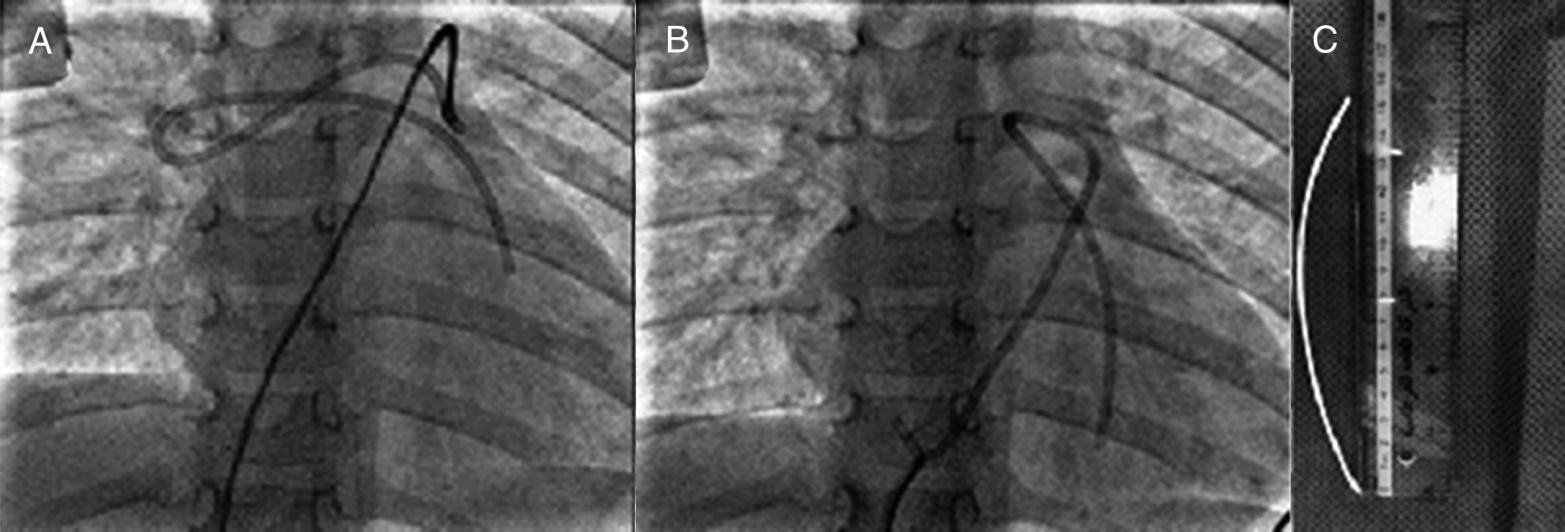
(A) Antero-posterior fluoroscopic view showing embolised central venous catheter in the right ventricle outflow tract and folded in the pulmonary artery. The tip of the catheter in the left pulmonary artery was holed by snare. (B) A fluoroscopic view showing the embolised catheter during retrieval and the snared tip of the dislodged catheter at the level of inferior vena cava during retrieval. (C) The extracted catheter (16 cm long) after retrieval.

This study evaluated the referral preference of PHCP in two tertiary care centres in Riyadh, Saudi Arabia, for management of paediatric patients with embolised foreign bodies originating from central lines. According to our knowledge, no such reports have been published from the region about this subject.

## Methods

A short questionnaire was distributed to PHCP (*n*=128) in two tertiary care centres between September 2011 and February 2012.

Paediatric trainees beyond the 3rd year of their residency programme, paediatric fellows, and consultants of all paediatric subspecialties, including paediatric surgeons, were included. All participants received the questionnaire from the paediatric department secretary during their morning handover meetings. The filled questionnaires were collected by the same secretary.

In addition to the personal data, the questionnaire included three main questions: the number of patients seen per year with central lines, experience of complications with central line, and the specialty to which they prefer to refer cases of dislodged or broken central lines. Six different options of referral specialties were offered: vascular surgery, paediatric surgery, radiology, paediatric cardiology, cardiac surgery, and paediatric intensivist (see Appendix for detailed questionnaire). Incompletely answered questionnaires were excluded from the analysis.

## Results

The questionnaire was distributed to 128 medical professionals. A total of 101 (79%) participants answered the questionnaire. Incomplete answers (*n*=14) were excluded, leaving 87 questionnaires for analysis. The grades of responders were senior consultants 18%, junior consultants 38%, and senior residents 43%. Of the responders, 39% saw more than 30 central lines per year and 39% experienced dislodgment complications. Referral to paediatric cardiac surgery or paediatric general surgery for removal of an embolised foreign body was the preference of 82% of the PHCP.

## Discussion

It is important for PHCP dealing with central lines to become familiar with transcatheter retrieval of fragmented ones. Over the last decades, the rapid development and wide application of minimally invasive and interventional techniques had proved that transcatheter retrieval of intracardiac and intravascular foreign bodies to be a standard, safe, and easy method (4–6).

Most of the PHCPs in this study (>80%) did not send their patients for transcatheter removal of the intravascular dislodged foreign bodies. These PHCPs were probably either not aware of this less invasive approach or of its local availability. This possibly indicates the need to improve knowledge of non-surgical methods of treatment and of their availability. It also highlights the importance of continuous professional development on the least invasive management options and the importance of better communication between departments within the same hospital as well as between related specialties at different hospitals.

The selected hospitals were chosen out of convenience and not at random. We chose to use a short questionnaire to make it simple and more likely to be answered, though many other questions might have been included. The results clearly show referral to surgery in most cases. It is now important to examine the level of knowledge about transcatheter retrieval of intravascular foreign bodies.

## Conclusions

Paediatric health care providers in the selected hospitals prefer to send their patients with embolised intravascular foreign bodies for surgical intervention. Further training is needed to promote knowledge of the existence of the percutaneous approach to improve patient care and reduce the number of unnecessary surgeries.

## Appendix

Intravascular or intracardiac foreign body removal

Date …………….

Speciality……………………. Subspecialty…………………….

Grade…………………………. Department…………………….

**Q 1**: What is the number of patients you see with central line (e.g. portacath, central venous line, umbilical catheter) per year? Choose one of the below.

- 1–10
- 10–20
- 20–30
- More than 30

**Q 2**: Did you have experience with fragmentation and dislodgement of catheter line. Choose one of the below

- Yes
- No

**Q 3**: In case of dislodged broken central line catheter to whom will you refer for removal. Choose one of the below.

- Vascular surgery
- Paediatric surgery
- Radiology
- Paediatric cardiology
- Cardiac surgery
- Paediatric intensivist

We really appreciate your participation in this project.
